# Primary Gastric Signet Ring Cell Carcinoma or Metastatic Lobular Carcinoma With Signet Ring Cells: A Diagnostic Challenge

**DOI:** 10.7759/cureus.42502

**Published:** 2023-07-26

**Authors:** Nada Akouh, Nassira Karich, Anass Haloui, Asmae Aissaoui, Abdelkrim Zazour, Zahi Ismaili, Amal Bennani

**Affiliations:** 1 Pathology Department, Mohammed VI University Hospital, Faculty of Medicine and Pharmacy of Oujda, Mohammed First University, Oujda, MAR; 2 Gastroenterology and Hepatology Department, Mohammed VI University Hospital, Faculty of Medicine and Pharmacy of Oujda, Mohammed First University, Oujda, MAR

**Keywords:** diagnostic pitfall, rare entity, lobular carcinoma, signet ring cell carcinoma, breast carcinoma, gastric metastasis, appropriate treatment

## Abstract

Histologically, cells having vacuolated cytoplasm rich in mucin and pushing the nucleus to the periphery are indicative of signet ring cell carcinoma. This condition often affects the digestive system. On the other hand, it is a very uncommon subtype of invasive lobular breast carcinoma, with a higher probability (more than invasive breast carcinoma of no special type {IBC-NST}) to migrate to the stomach, spleen, urinary tract, and uterus.

As with other metastatic carcinomas of breast origin, metastatic signet ring cell carcinoma of the breast is often treated with systemic therapies such as chemotherapy or hormonal therapy. However, surgical resection and eventual perioperative chemotherapy are usually recommended in case of primary gastric ring cell carcinomas that are non-metastatic. As a result, misdiagnosis might result in unneeded gastrectomy and chemotherapy, which would result in considerable mortality and morbidity.

We report a case of mammary lobular carcinoma with signet ring cells metastatic to the stomach, a variant rarely described and challenging to distinguish from primary gastric signet ring carcinoma.

## Introduction

Invasive lobular carcinoma is the second most common type of breast cancer (8% to 14%). It is a special subtype, distinguished by discohesive tumour cells organized in single files or as independent single cells. It results in a diffuse infiltration in the breast, and when metastatic, it involves a similar infiltrative diffusion, involving the gastrointestinal tract, peritoneum, retroperitoneum, bone marrow, meninges, and uterus [1]. It has a particular tendency to involve the gastric wall diffusely, and on histological examination, this can easily lead to retaining a false diagnostic of primary poorly cohesive gastric cell carcinoma, especially when it is lobular carcinoma in its signet ring cell variant [1].

Given the immense interest in adequately differentiating between the two entities histologically and immunohistochemically, this case study is going to help us to highlight the essential features that differentiate a primary site apart from a secondary location with the fundamental aim of appropriate treatment.

## Case presentation

A 54-year-old female patient with a history of Scarff-Bloom-Richardson (SBR) Grade II infiltrating lobular left breast carcinoma was diagnosed initially on breast microbiopsy and confirmed on a lumpectomy specimen with homolateral axillary curage performed in October 2018. Surgical resection margins were clean, and lymph node dissection was found 12N+/18N. Subsequently, the patient received 18 cycles of chemotherapy, the last of which was in May 2019, and 35 sessions of radiation therapy, the last of which was in August 2019. She was undergoing hormone therapy with letrozole 2,5mg at a rate of one tablet per day. The last mammography performed in June 2022 showed no suspicious lesions. The last positron emission tomography (PET)-Scan performed in October 2022 was also without abnormalities.

**Figure 1:**
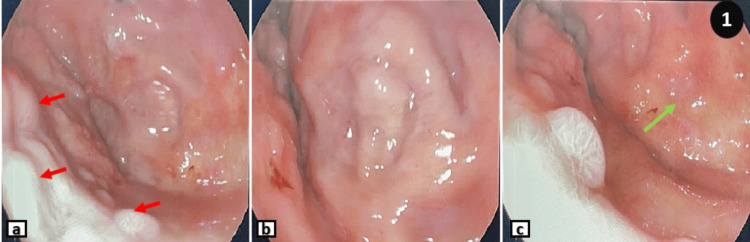
Endoscopic images of the gastric mucosa Endoscopic images showing gastric mucosa with multiple elevations (red arrows in image a) and erosions (the green arrow in image c).

The patient was admitted in November 2022 (three years after her last session of chemotherapy) to the hepatogastro-enterology department for exploration of a submucosal thickening of the entire gastric wall, measuring up to 9mm in thick in places. This thickening was discovered accidentally during a thoracoabdomino-pelvic CT scan performed as a part of the check-up. It did not reveal any breast lesion suspicious of malignancy. An endoscopic exploration by oeso-gastro-duodenal fibroscopy was performed, showing a congestive antro-fundic gastric mucosa with multiple scattered elevations of variable size, some of which were depressed in the center and others ulcerated on the surface (Figure 1). Multiple biopsies were performed, and the specimen was processed for histopathological study.

**Figure 2:**
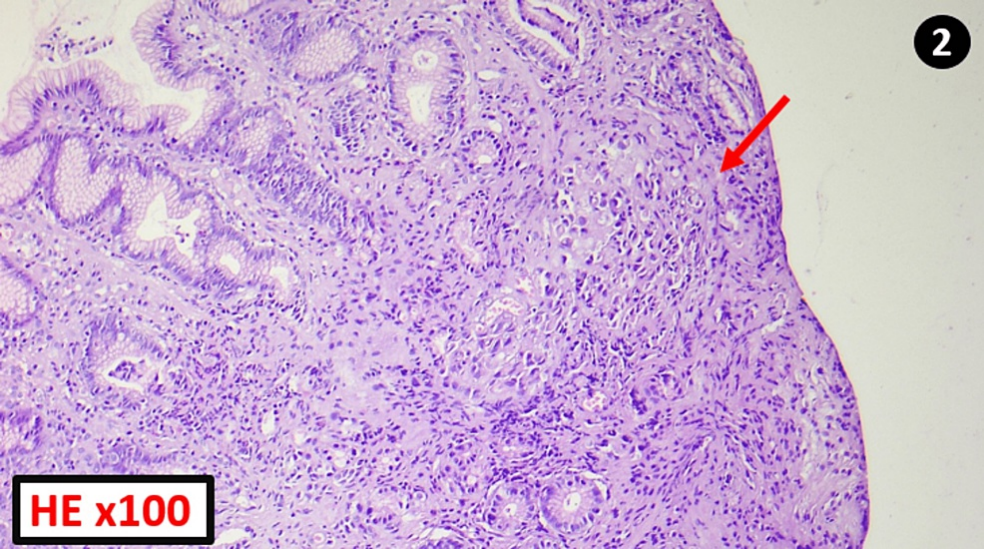
Histopathology image HE: Hematoxylin and Eosin Stain Gastric mucosa is extensively infiltrated by diffuse tumor proliferation (red arrow).

**Figure 3:**
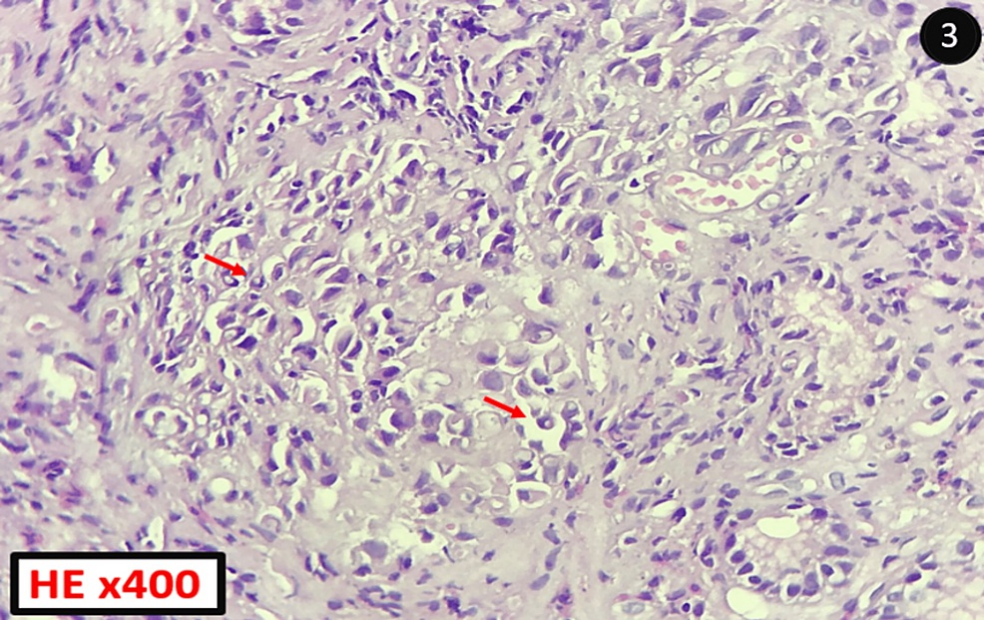
High-resolution histopathology image HE: Hematoxylin and Eosin stain The tumor cells are poorly cohesive, showing a signet-ring appearance (red arrows).

The histological examination of the received fragments found an antro-fundic mucosa with a proliferation of loosely cohesive cells arranged in diffuse sheets (Figure 2), with moderate cytonuclear atypia, hyperchromatic nuclei, and abundant cytoplasm, either eosinophilic or vacuolar, pushing the nucleus to the periphery (Figure 3). The presence of signet-ring cells in a gastric biopsy strongly suggests primary gastric disease; however, given the patient’s history, it was necessary to rule out a metastasis of a primary breast tumor; by immunohistochemical study, even in the absence of a breast lesion on clinical and radiological examination.

**Figure 4:**
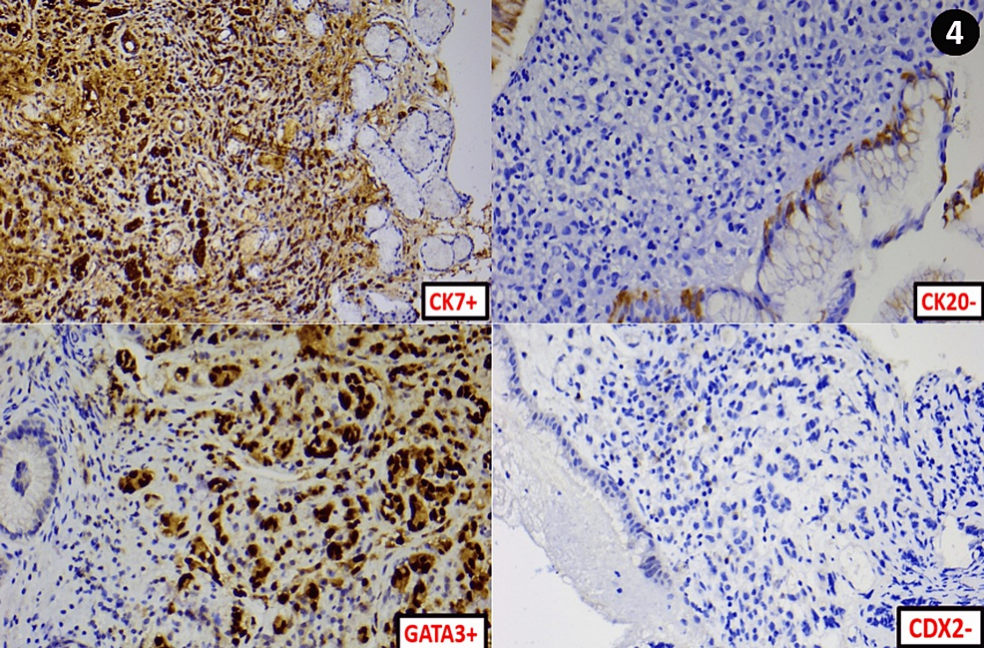
Immunohistochemical study images The tumor cells have a CK7+/CK20- profile, with diffuse GATA3 expression without CDX2 expression.

An immunohistochemical study was performed and showed positive labelling of the tumour cells by Cytokeratin 7 (CK7) and GATA binding protein-3 (GATA3). However, they did not express Cytokeratin 20 (CK20) and caudal type homeobox-2 (CDX2) (Figure 4).

**Figure 5:**
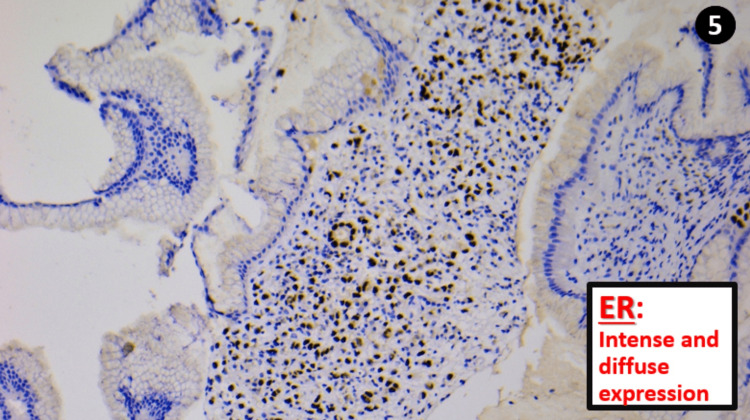
The tumor cells diffusely and intensely express estrogen receptors, confirming the mammary origin ER: Estrogen receptors

**Figure 6:**
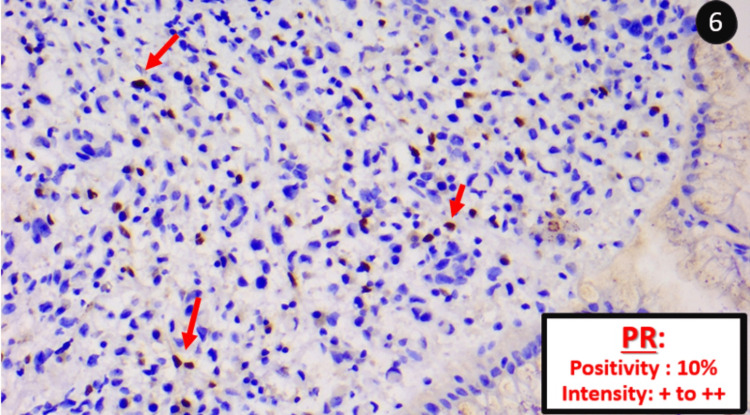
Tumour cells expressing progesterone receptors About 10% of the tumour cells express progesterone receptors (PR) (red arrows), with an intensity of + to ++, confirming the mammary origin.

Estrogenic hormone receptors (ER) were labelled in 95% of the tumor cells with an intensity of +++ (Figure 5), and progesterone receptors (PR) were expressed in 10% of the cells with an intensity of + to ++ (Figure 6).

The diagnosis of a secondary gastric localization of lobular breast carcinoma, in its signet-ring cells variant, was retained. The decision taken at the multidisciplinary consultation meeting between pathologists, gastroenterologists, visceral surgeons, and oncologists, in view of the histological and immunohistochemical appearance and the absence of any other metastatic site (recently explored), was to consider a total gastrectomy after chemotherapy sessions, alongside the hormone therapy the patient had been taking for four years. However, given the patient's non-consent to surgery, only chemotherapy (+hormone therapy) was administered.

## Discussion

A veritable diagnostic challenge is illustrated in this case; the presence of signet-ring cells in a gastric biopsy is extremely reliable with carcinoma of gastric origin; since it is often observed in the gastrointestinal system. However, it should be noted that breast origin should also be considered, especially if the patient has a history of breast neoplasia. If there is no known history, a clinical examination and mammography should be systematically performed.

Breast signet-ring cell carcinoma is a very uncommon condition that accounts for between 2% and 4,5% of all breast cacinomas [2] and has a poorer prognosis than other breast carcinomas. Immunohistochemistry is an important diagnostic tool to differentiate gastric and breast signet ring cell carcinomas. These tumours may be distinguished, in particular, by staining for ER, GATA3, and CDX2 [3]. While CDX2 positivity is more common in gastric tumours, ER and GATA3 positivity is more favourable in mammary origin (Table 1).

**Table 1:** Comparative table showing the differences between the immunohistochemical profiles of the stomach and the breast carcinomas References [3-6].

	CK7	CK20	GATA3	CDX2	ER
Gastric	Positive in 50% of cases	Positive in 40% of cases	Positive in 5% of cases	Positive in 20% to 90% of cases	Weak and focal expression possible.
Breast	Usually positive	Usually negative	Positive in 99% of cases	Very rarely positive	Intense and diffuse expression.

Initially, GATA3 positivity was an important indicator in determining breast origin. Recently, nevertheless, it was shown that stomach carcinomas, also, test positive in 5% of cases [4]. Gastric adenocarcinoma, on the other hand, exhibits CK7 positivity in 50% of cases and CK20 positivity in 40%. This variation makes it difficult to make distinctions between the two examined entities [5]. Moreover, CDX2 is variably positive (20% to 90%) in the stomach [5], but is also exceptionally positive in very rare cases of carcinoma of breast origin [6].

Finally, it is crucial to keep in mind that (while considering the coherence of the whole context) a focal and low-intensity positivity of estrogenic receptors does not exclude a primary gastric adenocarcinoma, but diffuse and high-intensity expression can [5].

## Conclusions

Treatment by chemotherapy or surgical resection depends heavily on the anatomopathological diagnosis (primary cancer *vs* metastasis). This makes this case study an invaluable source of information on the immunohistochemical differences between the two entities. In conclusion, the distinction between these two entities remains difficult. The GATA3/CDX2 binomial canorient the diagnosis. ER confirms it in favor of a mammary origin in case of intense and diffuse expression.
